# Activity in a Premotor Cortical Nucleus of Zebra Finches Is Locally Organized and Exhibits Auditory Selectivity in Neurons but Not in Glia

**DOI:** 10.1371/journal.pone.0081177

**Published:** 2013-12-03

**Authors:** Michael H. Graber, Fritjof Helmchen, Richard H. R. Hahnloser

**Affiliations:** 1 Institute of Neuroinformatics and Neuroscience Center Zurich, University of Zurich / ETH Zurich, Zurich, Switzerland; 2 Brain Research Institute, University of Zurich, and Neuroscience Center Zurich, University of Zurich / ETH Zurich, Zurich, Switzerland; 3 Institute of Neuroinformatics and Neuroscience Center Zurich, University of Zurich / ETH Zurich, Zurich, Switzerland; Utrecht University, The Netherlands

## Abstract

Motor functions are often guided by sensory experience, most convincingly illustrated by complex learned behaviors. Key to sensory guidance in motor areas may be the structural and functional organization of sensory inputs and their evoked responses. We study sensory responses in large populations of neurons and neuron-assistive cells in the songbird motor area HVC, an auditory-vocal brain area involved in sensory learning and in adult song production. HVC spike responses to auditory stimulation display remarkable preference for the bird's own song (BOS) compared to other stimuli. Using two-photon calcium imaging in anesthetized zebra finches we measure the spatio-temporal structure of baseline activity and of auditory evoked responses in identified populations of HVC cells. We find strong correlations between calcium signal fluctuations in nearby cells of a given type, both in identified neurons and in astroglia. In identified HVC neurons only, auditory stimulation decorrelates ongoing calcium signals, less for BOS than for other sound stimuli. Overall, calcium transients show strong preference for BOS in identified HVC neurons but not in astroglia, showing diversity in local functional organization among identified neuron and astroglia populations.

## Introduction

During motor behaviors and during working memory tasks, cortical premotor areas often show stereotyped sequences of neural activity [1]–[4]. Such neural sequences are thought to be shaped by both sensory inputs and experience-dependent plasticity mechanisms [5]–[9]. Thus, the spatial and temporal structure of sensory responses in (pre-) motor areas may provide clues for the functional organization of these areas.

Songbirds provide an outstanding opportunity to study sensory-motor learning on the basis of a clearly defined highly precise behavior. Control of motor output, i.e. song production, and processing of auditory input both require a distinguished set of brain areas. In HVC (used as a proper name), a pallial (cortical) motor area, responses to sensory inputs in single neurons tend to show remarkable selectivity for stimuli generated by motor output [10], [11], and strongly resemble responses recorded during generation of that motor output [12]. Three major types of HVC neurons have been identified: HVC_RA_ neurons projecting to the downstream motor nucleus robustus of the arcopallium (RA), HVC_X_ neurons projecting to the pallidal Area X in the anterior forebrain involved in song learning [13]–[15], and HVC interneurons. During singing, HVC_RA_ and HVC_X_ neurons fire a small number of short bursts of action potentials per song motif, whereas HVC interneurons fire more densely and with less temporal precision [16], [17]. In anesthetized birds, a fraction of HVC neurons respond to auditory stimulation [18], [19]. Interestingly all HVC neuron types respond stronger to the playback of the bird's own song (BOS) than to all other stimuli tested including white noise (WN), conspecific songs (CON), the BOS played backwards (reverse BOS, rBOS), and tutor song (TUT) [10], [20]–[22]. Individual HVC neurons can respond with astonishing temporal precision to the BOS, evidenced by individual spike bursts produced reliably in each trial and with millisecond precision [22]–[24]. Millisecond precision also exists in correspondence between singing-related and auditory-evoked activity [12], [25].

Multi-unit recordings revealed no evidence of auditory response topography. BOS responses are equally strong over the entire HVC of zebra finches [21]; and, responses to different songs in song sparrows (birds producing multiple songs) do not vary in strength across different HVC locations [26]. However, the spatial resolution provided by metal electrodes may not be sufficient to provide evidence for or against HVC local response clustering and auditory response topographies. Also, neurons may not be the sole cell type to provide clues about sensory-shaped motor sequences. Neurons are embedded in populations of supportive astroglia cells that often ensheath pre- and postsynaptic partners in a structure termed ‘tripartite synapse’ [27]. Astrocytic activity can be modulated by neuronal activity and sensory input [28], [29]. In songbirds, changes in astroglia populations can occur in synchrony with seasonal changes in song learning [30], but astrocyte signals and their relation with sensory inputs and neural signals have not been studied.

In head-fixed and urethane-anesthetized zebra finches we perform two-photon laser scanning microscopy of calcium activity in large populations of HVC neurons and astroglia. In HVC neurons we seek to characterize calcium activity in relation to action potential (AP) firing. We find good correspondence between calcium signals and juxtacellular electrical activity. In neurons and in astroglia we investigate auditory response properties and we study pairwise correlations of calcium activity and their dependence on both stimulus type and spatial separation. We find that overall there is diverse structure in baseline and auditory-evoked activity. Pairwise correlations fall off with distance between cells, and auditory stimulation has the effect of reducing correlations in a stimulus dependent manner.

## Methods

### Ethics Statement

All experimental procedures were approved by the Cantonal Veterinary Office of the Canton of Zurich, Switzerland (License numbers 75/2007 and 191/2010).

For two-photon calcium experiments we used 8 male zebra finches raised in our breeding colony. On the day of the two-photon experiment all birds were 90 days post hatch or older.

### Surgeries and labeling procedures

**Figure 1:**
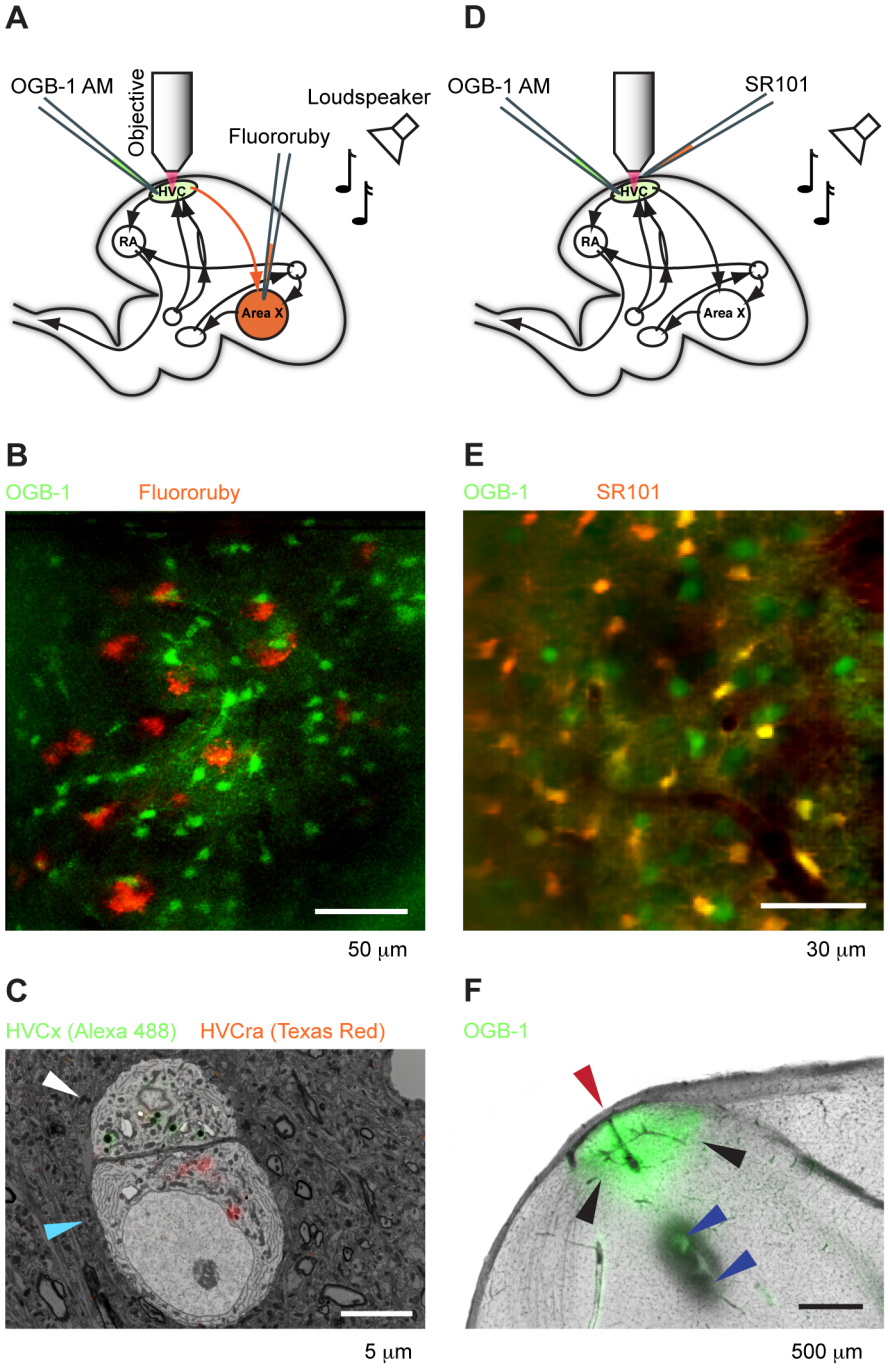
Calcium imaging of identified HVC cells. **A** Schematic sagittal view of the zebra finch brain with fluorescence labeling of unspecific HVC cells with OGB-1 AM (green shading) and X-projecting HVC neurons with retrograde tracer fluororuby (orange shading in Area X). We played back auditory stimuli while imaging calcium activity in OGB-1 filled cells using a two-photon microscope (red beam). **B** Maximum projection onto xy plane of a 3D image stack from an HVC site at 180–220 µm imaging depth with retrogradely labeled HVC_X_ neurons (red) and OGB-1 filled cells (green). **C** Overlay of light microscopic and electron microscopic imagery of an HVC_X_ soma (white arrow) and an adjacent HVC_RA_ soma (blue arrow). Retrograde tracer dye is located mainly in organelles in the cytosol near the nucleus (red: Texas red injected into Area X; green: Alexa 488 injected into RA). **D** In different experiments we labeled astroglia with SR101 (orange) instead of injecting fluororuby into Area X. **E** Average projection of an HVC imaging site with SR101-filled cells (green) and OGB-1 filled cells (red). Double-labeled cells appear yellow. **F** Sagittal histological section (anterior right, dorsal up) showing the remains of the calcium indicator OGB-1 AM in HVC (green shading, black arrows). Visible are also the pipette/electrode track (red arrow) parallel to the optical axis of the two-photon microscope and two small electrolytic lesions (blue arrows) at 1.5 and 2 mm depth made at the end of the experiment.

For retrograde labeling we injected 3 birds with a red dextran conjugated tracer dye (Fluororuby/Texas Red 10′000 MW, Invitrogen, Molecular Probes, dissolved in distilled water to achieve maximum concentration as specified by the vendor), Figure 1 A–C. Birds were anesthetized with isoflurane (1–5%) and retained in a stereotactic device at an angle of 65 degrees of the plate forehead to horizontal. We removed the feathers and applied lidocaine locally before making an incision into the skin. A small craniotomy was drilled into the skull using a dental drill. We stereotactically targeted Area X with a pipette filled with retrograde tracer and used a Picospritzer to pressure eject small volumes (∼500 nl) of tracer dye. We closed the wounds with medical glue and let the bird recover in isolation for a few days. Birds typically recovered well and often sang the day after the tracer injection.

On the day of the two-photon microscopy experiment, we injected birds intramuscularly with 2–3 dosages of 30 µl of 20% urethane dissolved in Ringer solution, we waited roughly 30 minutes between injections. Right after the second urethane injection we started the surgical intervention by additionally anesthetizing the bird with isoflurane to bridge the slow onset of urethane anesthesia. Isoflurane anesthesia was induced with 3–5% isoflurane in humified air for approximately 2 minutes until birds' breathing rate was stable around 60 bpm; thereafter we maintained peri-surgical anesthesia with 1–2% isoflurane. After the surgical intervention, before imaging, isoflurane administration was ceased. We used custom-made head holders together with the stereotax to avoid the usage of ear bars (such bars may have detrimental effects on hearing). The bird's head was placed in the head holder at an angle of roughly 80 degrees of the plate forehead to the horizontal. We removed the feathers on the posterior part of the head and applied Lidocaine gel locally to the scalp before making an incision into the skin. We drilled a small hole into the first bone layer of the skull above left HVC (based on stereotactic coordinates). We then attached a metal headplate to the bird's head using dental cement. We removed the inner bone layer right above HVC and perforated a tiny part of the dura with a syringe needle to allow easy penetration with an injection pipette. We bolus loaded the synthetic calcium indicator Oregon Green BAPTA-1 (Figure 1 A, B & D–F) masked with an acetoxymethyl ester for cell membrane penetration (OGB-1 AM, Molecular Probes, Invitrogen) according to a recently established protocol [31], [32] as follows: We prepared the fluorophore injection solution on the day of the experiment prior to the surgery. 1–3 µl of 20% Pluronic F-127 (BASF) dissolved in Dimethylsulfoxid (DMSO) was used as a solvent for 50 µg of OGB-1 AM (Molecular Probes, Invitrogen). We complemented the solution with calcium-free Ringer solution to 40 µl in total, leading to a solution of about 1 mM OGB-1 AM. We lowered a glass pipette filled with the solution into HVC and in a slow process of about 10 minutes we injected a few hundred nanoliters at a pressure of about 0.3 bar.

To label astrocytes (n = 2 birds) we applied a solution of 50 µM Sulforhodamine 101 (SR101) in Ringer directly onto the surface of the brain (Figure 1 D) for about 10 minutes and then flushed the brain with Ringer solution. SR101 labeled cells showed typical characteristics of glia cells: They tended to more brightly stained by OGB-1 AM and were generally smaller than other cells. They often made contact with blood vessels and had clearly visible, short appendages, Figure 1 E.

We covered the craniotomy with 1% agarose (Sigma) in Ringer solution and clamped a cover glass to the headplate to stabilize the brain from heartbeat and breathing-induced motion.

### Histology

At the end of the experiment we killed birds with an overdose of Nembutal injected intraperitoneally, we removed the brain and fixed it in 4% PFA, and we cut sagittal sections of 80–100 µm thickness and imaged them in a light microscope. In the case of SR101 experiments we made electrolytic burns (10 s with 15 µA) below the site of OBG-1 injection before sacrificing the bird to verify the sites of calcium imaging in subsequent histology, Figure 1 F. Besides the remains of calcium indicator, histological sections typically revealed the track of the injection pipette, burns, and the retrograde tracer. We successfully recorded calcium activity while playing back auditory stimuli from n = 5 anesthetized and head-fixed zebra finches. We discarded data from 1 bird in which we found no cells that responded to stimulation with the BOS (Z-score ≥1). Among the reasons for not seeing auditory responses in this bird are sensitivity of calcium responses to damage caused by the brain surgery and absence of auditory responses in HVC of some birds. Experiments combining calcium imaging with the recording of extracellular voltage were done in n = 2 birds. The electron micrograph in Figure 1 C was prepared by Daniele Oberti using his correlative microscopy protocol [33]. It shows the retrograde tracers being located within organelles in the projection neuron soma.

### Song Recordings and Stimulus Generation

Prior to two-photon microscopy and tracer injection, birds were isolated, their songs were bandpass filtered in the range 0.3–13 kHz, digitized at 32 kHz sample rate, and saved to disk using custom recording software written in LabView (*National Instruments*). We used Matlab (*Mathworks Inc.*) to assemble several stimulus files, each composed of random permutations among 12 stimuli, in which 4 stimuli were randomly chosen from each of the 3 stimulus types: 1) bird's own song (BOS) stimuli of roughly 2–3 s duration, 2) BOS played back in reverse (rBOS) stimuli of similar duration, and 3) 3 s white noise (WN) stimuli. Stimuli were normalized to equalize their average sound intensity and were separated by 10 s silent intervals in all recordings that went into the population analysis and by 5 s in a few additional recordings. Overall the duration of one stimulus file was approximately 3 minutes. For each recording site we acquired imaging data in response to 1–3 stimulus files. We re-recorded the auditory stimulus files that we broadcast through a loudspeaker using a microphone and custom software written in LabView.

### Calcium Fluorescence Imaging in HVC

We used a custom built two-photon laser scanning microscope powered by 100 fs pulses at 870 nm wavelength of a Ti:sapphire laser (Spectra Physics). The laser beam passed through a 40x water-immersion objective (NA 0.8, Olympus) through which fluorescence light was collected as well. Imaging was controlled by the software package HelioScan written in LabView [34].

Dye uptake in the applied bulk loading protocol is known to be a slow process [31] that can take 0.5–3 h [35] depending on the species under investigation: In our experiments with zebra finches, fluorescence levels within cells reached stable values roughly 30–60 minutes after dye injection. Thereafter we started recording movies. While imaging we played back auditory stimuli through a loudspeaker positioned centrally ca. 20 cm in front of the bird. We placed a microphone right next to the bird's beak to record the auditory scenery, allowing for post-hoc detection of disturbing sounds and exclusion of affected recordings. During imaging, there was a constant faint high-frequency tone generated by the scanning mirrors of the microscope.

HVC is situated superficially in the posterior pallium. It is covered by a layer of parahippocampus and separated from it by the lateral ventricle (LV). The thickness of parahippocampus over HVC decreases from medial to lateral and from anterior to posterior. Whereas medial anterior HVC can be obstructed by a few hundred micrometers of tissue, only a thin layer of a few tens of micrometers separates lateral HVC from the brain surface. To avoid deep tissue imaging, we imaged mainly in lateral parts of HVC, roughly 2.5 mm lateral of the sagittal sinus. When we moved the optical plane from the surface to increasing depths (along z direction) we observed lack of OGB-1, staining when passing through the LV. As soon as the focal plane moved out of the LV into HVC, retrogradely traced X-projecting HVC neurons (HVC_X_ neurons) could be seen. We imaged from regions in which we could find either well-labeled astroglia or HVC_X_ neurons (the latter are known to fire at 1.5+/−0.4 Hz under urethane anesthesia [24]). We acquired frame scans of 128×128 pixels with a frame rate of 7.81 or 3.91 Hz and stored them to disk.

### Electrophysiology

**Figure 2:**
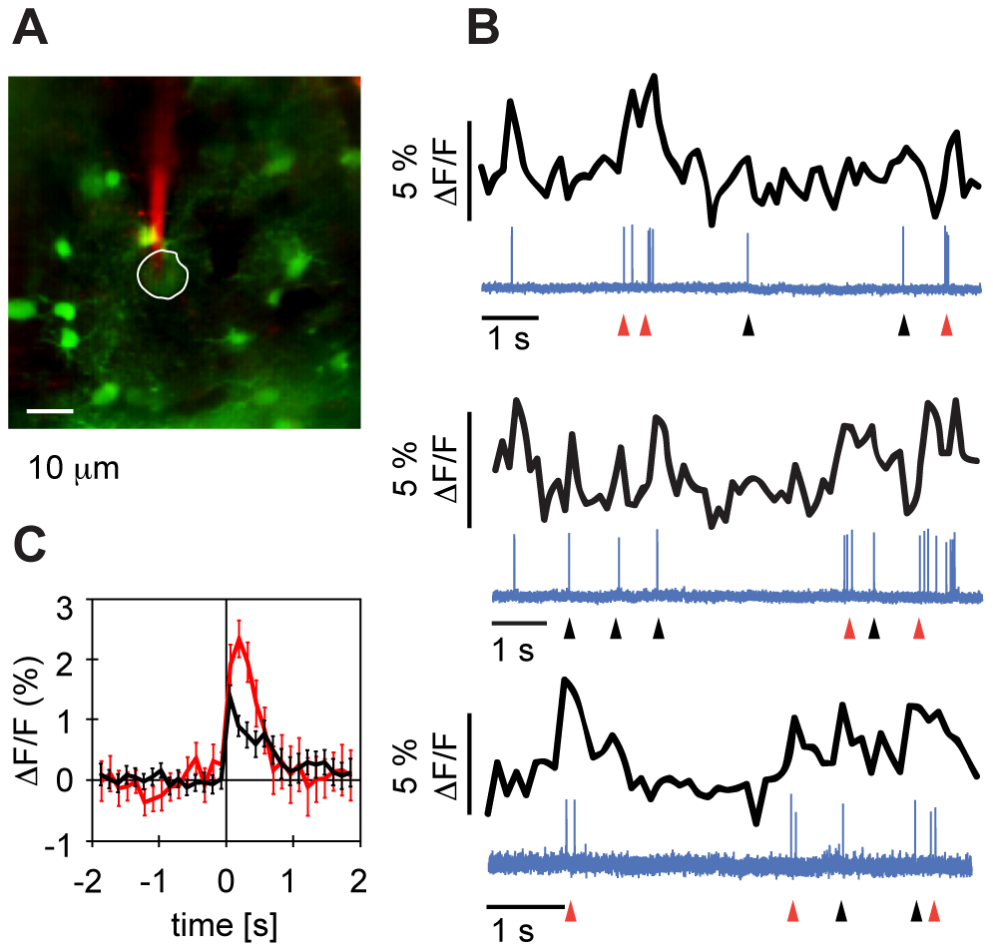
Action potentials are associated with fast calcium transients. **A** Targeted juxtacellular recording of membrane potential from an unidentified HVC neuron (circumscribed in white) using a patch pipette containing 50 µM Alexa 594 (red). **B.** Three example traces of simultaneously recorded calcium signals (black) and juxtacellular membrane potential (blue, arbitrary units) of the neuron in A. Short increases in firing rate (low-frequency bursts, red arrows) but also single isolated spikes (black arrows) were associated with transient increases in fluorescence of up to 5%. **C** Event-triggered average (ETA) ΔF/F signals of the cell in **A** and **B** reveal average fluorescence transients of 1.4% for single spikes (black) and of 1.9% for low-frequency bursts (red, burst width  = 200 ms) in the first bin after the event. The error bars indicate the standard error of the mean.

We made targeted juxtacellular recordings, Figure 2 A, B, under the two-photon microscope using borosilicate glass pipettes of impedances in the range 2–5 MΩ, filled with ringer solution and 50 µM Alexa 594 (Molecular Probes, Invitrogen) to see the pipette under the microscope. Electrical signals were amplified with an Axoclamp 2-B amplifier (Axon Instruments, Molecular Devices) in current clamp mode, digitized at 32 kHz (85 kHz in one case) sample rate, and saved to disk using custom written software in LabView.

### Data Analysis

All data were analyzed using custom software written in Python using *scipy* and *numpy* and visualized with *chaco* and *traits* (Enthought Inc.).

ROIs: Regions of interest (ROIs) were defined based on OGB-1 contrast in average projections of movie stacks. ROIs were manually selected. HVC_X_ neuron ROIs were such that they fully circumscribed the region of tracer fluorescence, known to emanate mainly from organelles near the nucleus, see Figure 1 B, C. ROIs of unidentified cells and astroglia were chosen to contain the region of high OGB-1 contrast. We chose neuropil ROIs in regions of roughly constant OGB-1 intensity and not obviously containing ROIs of other types. To assess background fluorescence we also chose ROIs in dark areas, mainly blood vessels, from which we calculated the time and space-averaged fluorescence signal 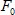. To compute the calcium signal in all ROIs corresponding to cells and neuropil, we first subtracted 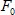 from the spatially averaged fluorescence signal, resulting in 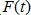. Based on 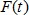 within a ROI we calculated the relative percentage changes, i.e., the calcium transient signal 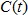 as
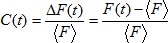
where 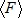 represents the time average of 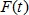 over the entire recording session. In the end, to remove very slow drifts, we high-pass filtered 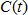 using a fast Fourier transform with cutoff frequency 0.06 Hz.

Event-triggered average: There were large differences between sampling rates of fluorescence recordings and electrophysiological recordings. We computed the event-triggered average calcium signal in Figure 2 C by setting the sample times of the fluorescence traces to the time points at which the last line of the ROI was scanned. Fluorescence samples were then binned into 128 ms bins with the spike/burst event set to the origin, and averaged.

### Stimulus responses and selectivity

We computed the calcium response 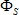 to a given stimulus by averaging the calcium signal 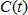 over the time period from stimulus onset to stimulus offset. The Z-score response to a stimulus was defined as the difference between 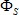 and a baseline response 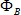 of mean calcium signal in a 4 s window preceding the stimulus, averaged over all repetitions of a stimulus and normalized: 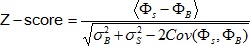
where 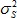 is the variance of calcium responses, 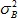 is the variance of baseline fluctuations, and 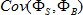 is the covariance (unbiased estimator) between stimulus and baseline. We assessed the selectivity of responses for one stimulus over another using the d′ measure according to which the average calcium response to stimulus S_2_ (averaged over stimulus repetitions) is subtracted from the average calcium response to stimulus S_1_:
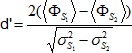



Note that that we excluded n = 32 highly responsive ROIs (n = 3 birds) from our population analysis that showed strong responses to low-frequency noise in the recorded stimulus files prior to stimulus onset (but following playback onset, mainly rBOS stimuli were affected). In these ROIs our estimate of baseline activity was contaminated (Z-scores were artificially diminished because of an elevated ‘baseline calcium signal’ elicited by the low-frequency noise). Among the ROIs excluded were 0 HVC_X_ neurons, 1 glia cell, 12 unidentified cells, and 19 neuropil ROIs. Their median Z-scores were: BOS 0.83, rBOS 0.08, and WN 0.46. The responsiveness for WN was clearly higher than for the set of regular ROIs, confirming the observation that overall these sites were more responsive to auditory stimuli/noise than average.

### Response correlations

We assessed correlations between calcium signals in pairs of ROIs in terms of the correlation coefficient (CC) between mean-subtracted calcium signals 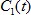 and 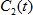:
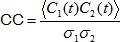



Where 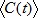 denotes time averaging of 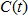 and where 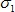 and 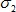 denote the standard deviations of the respective calcium signals. We separately computed CCs in time windows corresponding to auditory stimuli and to baseline activity.

Locking to population response: We computed a measure of population synchrony given by the correlation coefficient (CC) between the calcium signal (as a function of time) in a given ROI and the simultaneously recorded average signal in all other ROIs of the same type (estimate of population response). We calculated CCs for different stimulus types during presentation of auditory stimuli and during baseline periods.

Distance effect: To assess pairwise CCs as a function of distance between ROIs, we binned distances between ROI centers into 20 µm bins and averaged CCs over ROI pairs at equal distances (both for stimulus and baseline periods). We explored changes of bin size by a factor of two but did not find qualitative differences to the reported results.

We fitted the pairwise CC data as a function of ROI distance by an exponential curve of the form 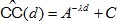, where 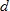 represents the distance between ROI centers. The fit of parameters 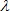, 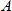, and 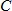 was computed using a downhill simplex algorithm. In the case of HVC_X_ neurons and WN stimulus conditions, we excluded three outliers from the dataset before fitting. These data points were less than 10 µm apart and had CCs >0.25.

### Auditory response dynamics

**Figure 3:**
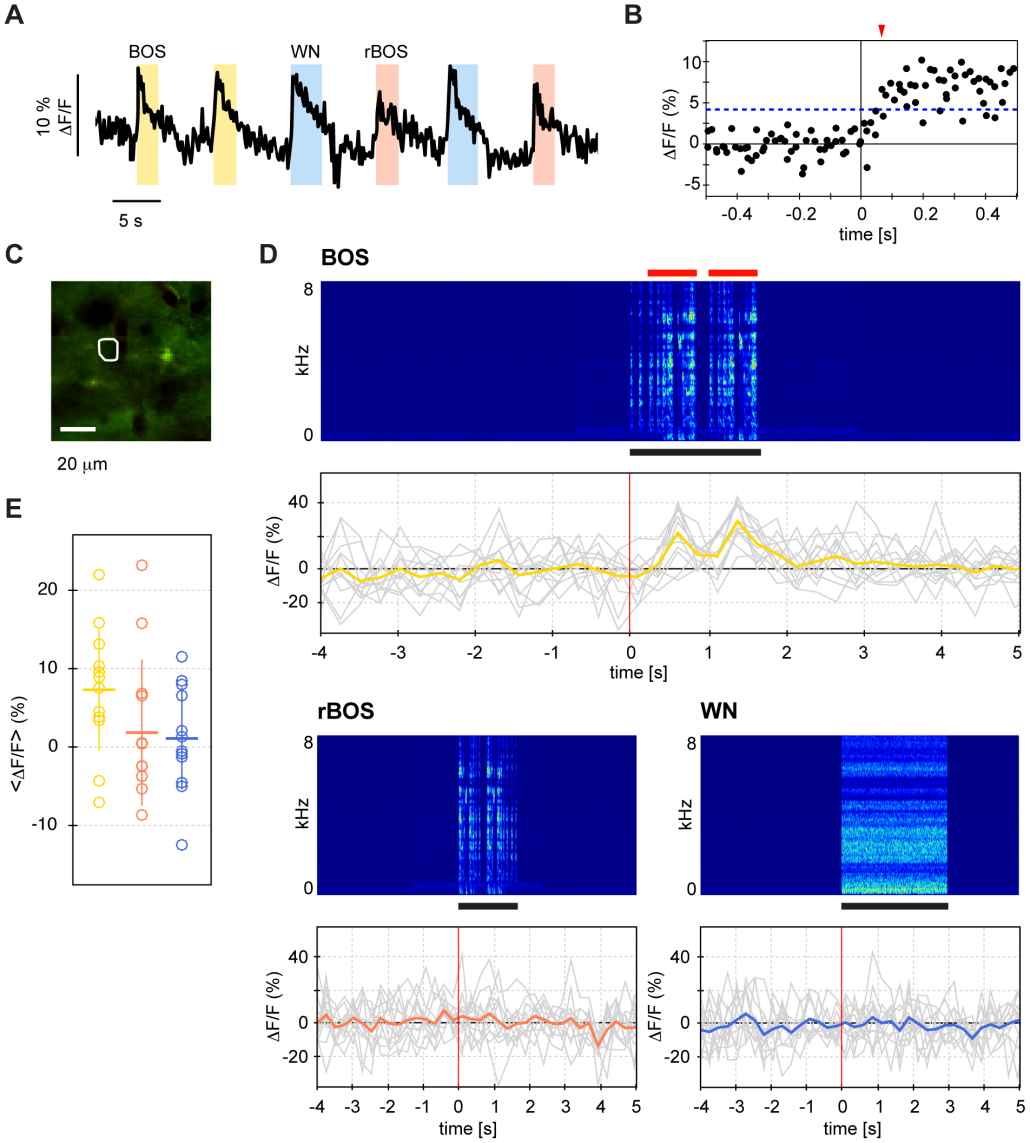
Time scales of auditory responses. **A** Example signal of an unidentified HVC cell with fast responses to sound onsets. All stimuli (blue: WN, yellow: BOS, red, rBOS) elicit a reliable calcium transient. **B** The ΔF/F samples aligned to stimulus onset (time origin) reveal a response onset (red arrow) of 66 ms; response onset was defined as the first data point above a threshold (blue dashed line) of three ΔF/F standard deviations estimated during baseline. **C** Average image of an acquired movie stack with the ROI of an undetermined cell (not filled with SR101) encircled in white. **D** Auditory responses of the cell in C. On top of each response panel we show the sound spectrogram recorded during calcium imaging (red: high signal amplitudes, blue: low amplitudes), stimuli are indicated with black lines; below are the individual ΔF/F responses in gray and the mean responses in color. This cell exhibits a ΔF/F response peak during playback of each of the two BOS motifs (indicated by red horizontal bars shown above the spectrogram). **E** Baseline-subtracted mean responses of the cell in C and D. The cell responds most strongly to BOS: Z-score BOS  = 0.90, Z-score rBOS  = 0.19, Z-score WN  = 0.1. The horizontal colored bars indicate the Z-scores and the vertical colored lines the standard deviations.

Auditory responsiveness varied widely among ROIs, even among ROIs of a given type. Only very few cells were strongly driven by auditory stimulation (visible in single-trial responses). An exceptionally responsive (non-identified) auditory cell is shown in Figure 3 A. This cell displayed (ΔF/F) transients to sound onsets on the order of 10%. In this cell we estimated the latency of calcium responses to sound onsets. The presentation of auditory stimuli was not synchronized with acquired frames in the microscope, therefore the time lags between stimulus onset and acquired fluorescence samples in the ROI were widely distributed across stimulus presentations. To make use of this diverse sampling offset, we time stamped the fluorescence samples by the times at which the scanning beam reached the first line beyond the ROI. We defined the response onset latency as the time lag of the first fluorescence sample that exceeded pre-stimulus baseline fluorescence by 3 standard deviations. In the cell in Figure 3 A the response onset latency, Figure 3 B, was 66 ms, which is shorter than the typical song syllable duration of 100–150 ms. Hence, our imaging approach allowed us in principle to associate activity in single neurons to acoustic events on a syllabic timescale. Furthermore, we noted that in some cells such as the unidentified cell in Figure 3 C–E, calcium transients accurately mirrored the song structure at sub-second resolution: The fluorescence signal in question displayed peaks that were time-locked to the BOS motif (the motif is a stereotyped roughly 1 s long song pattern composed of several syllables that birds repeat 2–5 times per song). Thus, OGB-1 fluorescence signals reflected periodicity in the auditory input on the time scale of several hundred milliseconds.

## Results

To study auditory-evoked cellular activity in HVC we injected the synthetic calcium indicator Oregon Green BAPTA-1 (OGB-1 AM) in urethane-anesthetized zebra finches, unselectively staining populations of both neurons and glia cells [31], [32]. To identify a neuronal subtype in HVC we retrogradely labeled HVC_X_ neurons by injection of fluorescent tracers into Area X, and to identify astrocytes we applied a known marker for astroglia, Sulforhodamine 101 (SR101).

### HVC neuron spikes are associated with transient increases in OGB-1 fluorescence

In rats, single action potentials are accompanied by OGB-1 fluorescence changes of up to 10% and multiple action potentials in short succession linearly add up to the detected change in fluorescence [36]. To investigate the relationship between OGB-1 fluorescence and spiking activity in HVC neurons, we imaged OGB-1 loaded neurons while juxtacellularly recording their membrane potential. Single action potentials did not consistently lead to detectable OGB-1 fluorescence changes ΔF. Low-frequency bursts (inter-spike interval <200 ms) however often co-occurred with ΔF/F transients of 2% or more, Figure 2 A–C. For the cell shown in Figure 2 A, B, the mean ΔF/F signal in the first 240 ms following (low-frequency) bursts was 50% larger than the mean ΔF/F signal following single spikes (T-test, p = 2*10^-4^, n = 53 single spikes, n = 15 bursts). On average, the spike-triggered average ΔF/F (combining single spikes and low-frequency bursts) was 1.5% (range 0.7–3.1, n = 5 cells in 2 birds). Transients had a sharp rise time and decayed roughly exponentially with average decay constant 0.94 s (range 0.47–1.87 s, n = 5 cells in 2 birds). These data confirm the positive correlation between calcium transients and neuronal firing, even though the spike-associated calcium increases we found were smaller than in rodents [36], [37] but of comparable size to transients in the zebra fish [38].

### Auditory responsiveness and stimulus selectivity

We broadcast random sequences of BOS, rBOS, and WN stimuli played through a loudspeaker at physiological sound amplitudes and with intermittent 5 or 10 s pauses. We analyzed calcium transients (ΔF/F) using standard measures in songbird auditory physiology research, i.e., *responsiveness* in terms of the Z-score of stimulus-evoked calcium signals (signals relative to baseline, normalized to units of standard deviation) and *selectivity* in terms of the psychophysical d′ measure (the normalized difference between mean calcium signals in response to two different stimuli). We imaged from different regions of interest (ROIs) including HVC_X_ neurons, astroglia, unidentified cells, and neuropil.

**Figure 4:**
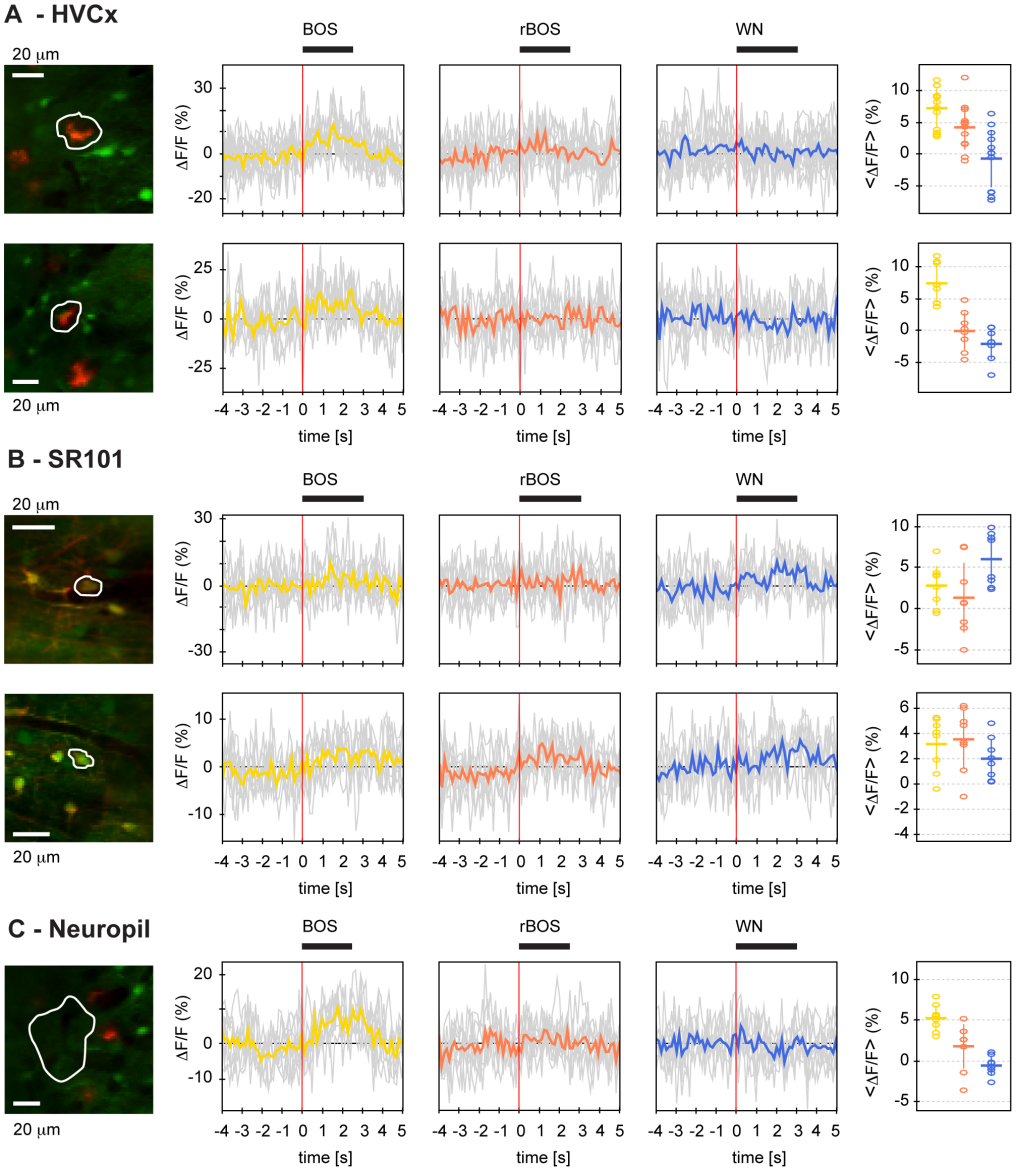
Auditory responses in different identified HVC cells and neuropil. **A–C** Left: Mean images of acquired movie stacks with the ROIs encircled in white. Middle: Marked on top are auditory stimuli (black horizontal bars); below are the individual ΔF/F responses (gray) and the mean responses (color). Right: Baseline-subtracted ΔF/F responses averaged over stimulus durations (WN shown in blue, rBOS in red, and BOS in yellow). Legend as in Fig. 3 E. **A** Response panels of two HVC_X_ neurons, with the neuron on top responding more to BOS (Z-score  = 0.98) than to rBOS (Z-score  = 0.58); the WN stimulus slightly suppresses OGB-1 fluorescence (Z-score  = −0.25). The neuron is BOS-selective: d′ BOS-WN  = 1.42, d′ BOS-rBOS  = 0.40, d′ WN-rBOS  = −1.10. The HVC_X_ neuron below shows even stronger BOS selectivity, d′ BOS-rBOS  = 3.33 and d′ BOS-WN  = 4.61. This neuron is neutral in its preference for WN vs. rBOS (d′ = 0.17). **B** Auditory responses in two cells labeled with SR101, i.e. presumptive astrocytes. The first cell responds strongest to WN (Z-score  = 1.86), but also to BOS (Z-score  = 1.09), whereas rBOS elicits only a weak response (Z-score  = 0.30). The second cell responds about equally to all auditory stimuli with Z-scores in the range of 1.2–1.5. **C** Neuropil ROI showing large response to BOS (Z-scores for BOS  = 3.19, for rBOS  = 0.61, and for WN  = −0.45).

**Figure 5:**
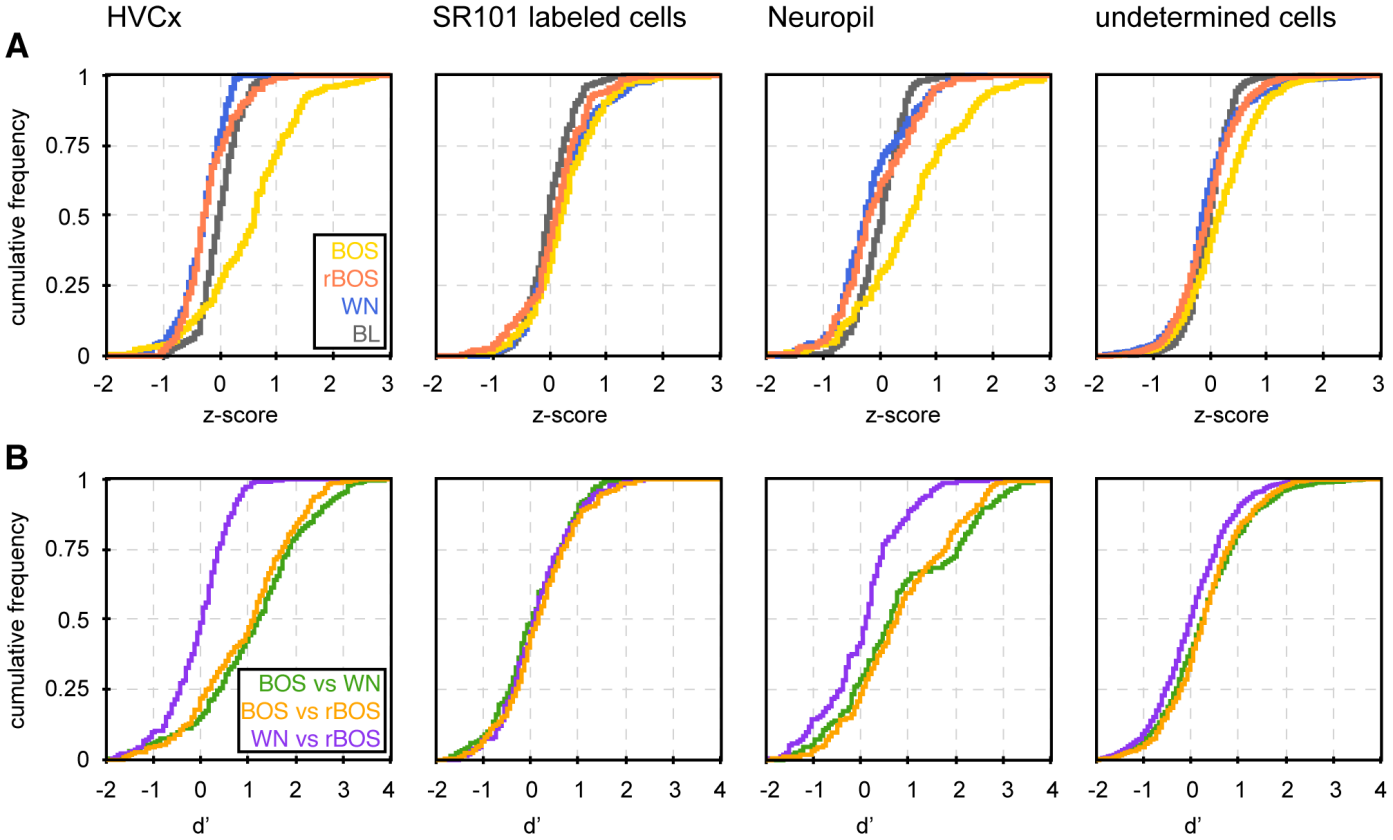
Auditory responses display preference for BOS in HVC_X_ neurons but not in astroglia. **A** Cumulative distributions of Z-scores in different ROI types and for different auditory stimuli. HVC_X_ neurons are strongly excited by BOS (yellow) but weakly inhibited by WN (blue) and rBOS (red). A very similar finding applies to neuropil, but not to astroglia in which auditory stimulation only elicits weak responses. The Z-score cumulative distributions during baseline periods (black) are antisymmetric and centered around zero, they depict the range of Z-scores associated with baseline signal fluctuations. **B** Distribution of d′ values in different ROI types and stimulus pairs. HVC_X_ neurons clearly prefer BOS over rBOS (orange, median d′ = 1.13) and BOS over WN (green, median d′ = 1.25), but display no differential responses to WN vs. rBOS (purple, median d′ = 0.05). A similar behavior is seen in neuropil, though there the preferences for BOS over rBOS (median d′ = 0.76) and BOS over WN (median d′ = 0.67) are less pronounced than in HVC_X_ neurons. Astrocytes slightly prefer BOS over rBOS (median d′ = 0.18) but even less pronounced is their preference for BOS over WN (median d′ = 0.06).

Most cells were only weakly activated by auditory stimulation, Figure 4 A–C depicts typical examples of auditory responses in HVC_X_ neurons, astroglia, and neuropil. The median Z-scores over ROIs of a given type were quite small, in the range −0.25 to 0.6, Table 1 and Figure 5. In response to BOS, all ROI types showed positive median Z-scores (Wilcoxon signed-rank test, p<0.001), most pronounced in HVC_X_ neurons (median Z-score  = 0.63, p = 2.1*10^−16^) and in neuropil (median Z-score  = 0.56, p = 3.3*10^−10^). HVC_X_ neurons were suppressed by WN and rBOS (median Z-score  = −0.25 in each case, p<0.001), whereas in neuropil the median Z-score was only negative for WN (−0.23 p = 0.002) but not for rBOS. Z-score results were almost unchanged when summarized using the mean instead of the median (not shown).

**Table 1:** Median Z-scores with quartile range in brackets for different ROI types and stimulus types (BOS, rBOS, and WN) and for baseline periods (BL).

	N ROIs/N Birds	Z-score BL	Z-score BOS	Z-score rBOS	Z-score WN
HVCX	226/3	0.00 [−0.19, 0.27]	0.63 [−0.02, 1.10]	−0.25 [−0.47, 0.06]	−0.25 [−0.51, −0.01]
			p = 1.2*10−13	p = 3.3*10−8	p = 9.2*10−14
Glia	198/2	0.03 [−0.20, 0.32]	0.22 [−0.07, 0.62]	0.11 [−0.14, 0.42]	0.17 [−0.16, 0.51]
			p = 1.1*10−5		p = 0.003
Neuropil	155/5	0.04 [−0.20, 0.25]	0.56 [−0.07, 1.11]	−0.16 [−0.49, 0.46]	−0.23 [−0.56, 0.25]
			p = 8.4*10−8		p = 5.5*10−4
Undet. cells	659/5	0.01 [−0.26, 0.24]	0.17 [−0.20, 0.61]	−0.01 [−0.34, 0.28]	−0.10 [−0.34, 0.25]
			p = 3.2*10−13		

The reported P-values represent the probability that the median Z-score is equal to that of the BL distribution (paired Wilcoxon signed-rank test).

At a given site we kept imaging times short to avoid thermal tissue degradation and indicator bleaching. Consequently, we used few stimulus presentations (range N = 8 to 12 presentations per stimulus). We speculated that variability in Z-scores across ROIs could be largely due to the small number of stimulus presentations. To estimate the amount of Z-score variability due to small N, we computed baseline (BL) Z-scores by randomly pairing 4 s baseline periods with 3 s ‘baseline responses’ from recording periods with at least 3 s pauses between stimuli. The result was a standard deviation in BL Z-scores in the range of 0.3–0.4, suggesting that about half of the Z-score spread among different ROIs is due to the limited number of stimulus repetitions (see Table 1). Nevertheless, in all ROI types, the median Z-score for BOS was larger than the median Z-score for BL (paired Wilcoxon signed-rank test, p<0.001), thus confirming the overall preference for the BOS.

We assessed the fraction of BOS-responsive cells by a Z-score threshold 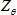 that was chosen to correspond to p<0.05, i.e., 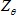 = 0.86 for N = 12 stimuli and 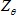 = 1.15 for N = 8 stimuli. We thus obtained a fraction of BOS-responsive HVC_X_ neurons of 31%, while 0% and 1% were responsive to WN and rBOS, respectively. We found that 28% of neuropil ROIs were responsive to BOS while 3% were responsive to WN and 5% to rBOS. Note that we suspect that these numbers are slightly biased to zero compared to numbers obtained in electrophysiology experiments (because of our choice of small N.

Our characterization of responses using Z-scores agreed with characterizations using d′ values, exemplified by the two HVCX neurons in Figure 4 C that showed preference for the BOS both with respect to WN and to rBOS (d′ BOS-rBOS  = 1.22 and 3.33, d′ BOS-WN  = 2.82 and 4.61). Overall, HVC_X_ neurons preferred BOS both over rBOS and over WN with positive median d′ = 1.1 for BOS-rBOS (Wilcoxon signed-rank test, p = 6.0*10^-14^) and positive median d′ = 1.3 for BOS-WN (p = 3.3*10^−11^) (see also Table 2 and Figure 5). Similarly, neuropil ROIs preferred BOS over rBOS (p = 3.2*10^−24^) and BOS over WN (p = 7.3*10^−26^). Glia cells, however, showed only significant preference for BOS over rBOS (p = 6.4*10^−4^) but no significant preference of BOS over WN (p = 0.24), see Figure 5.

**Table 2:** d′ values (median and [25%, 75%] quantiles) for different ROI types and pairs of stimulus types reveal preference for BOS over other stimuli in HVC_X_ neurons and in neuropil but not in glia.

	N ROIs/N birds	d′ bos-rbos	d′ bos-wn	d′ wn-rbos
HVCX	226/3	1.13 [0.15, 1.76]	1.25 [0.40, 1.89]	0.05 [−0.44, 0.39]
Glia	198/2	0.18 [−0.29, 0.69]	0.06 [−0.43, 0.65]	0.13 [−0.34, 0.63]
Neuropil	155/5	0.76 [0.06, 1.79]	0.67 [−0.10 2.12]	0.12 [−0.49, 0.49]
Undet. cells	659/5	0.29 [−0.20, 0.78]	0.27 [−0.28, 0.85]	−0.01 [−0.54, 0.55]

### Auditory stimulation decorrelates HVC_X_ activity

Two important advantages of two-photon imaging over electrophysiology are the abilities it provides to monitor activity simultaneously in large populations of identified cells (including glia cells) and to obtain precise information about their spatial distribution. For example, in auditory [39] and visual cortices [40] the pairwise correlation of activity decreases with increasing distance between cell pairs.

**Figure 6:**
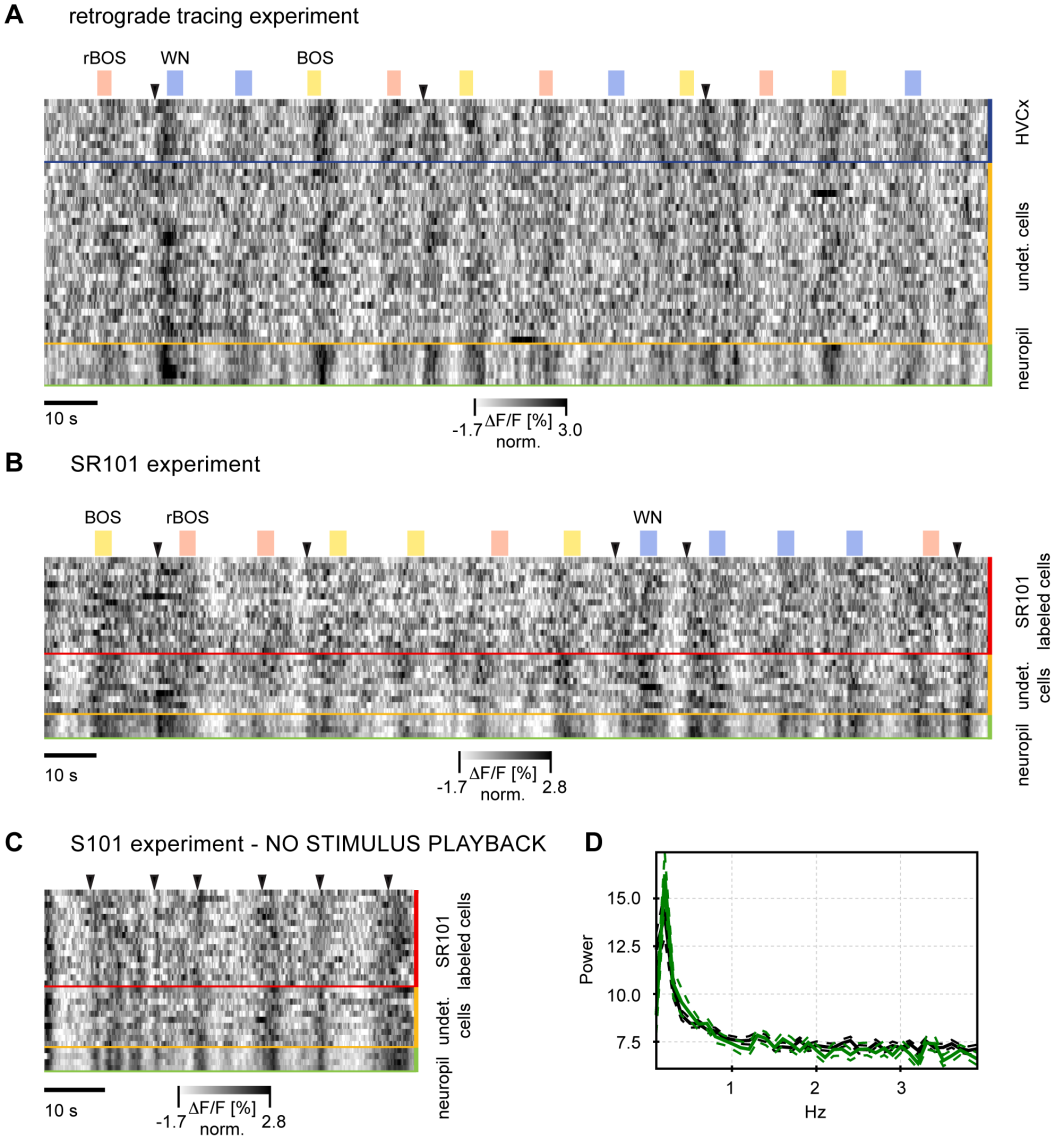
Stackplots of ΔF/F traces in ROIs of different types reveal synchronous activity. **A** Experiment with labeled HVC_X_ neurons, **B** experiment with labeled astroglia. **A** and **B**: Auditory responses modulate ongoing strong baseline signal fluctuations that are highly synchronized across the entire network. Many synchronized ΔF/F transients occur in the absence of auditory stimulation (black arrows). Stimulus playbacks are indicated on top. Each line represents the ΔF/F signal normalized by the standard deviation of that ROI, the darker the shading, the larger the ΔF/F values. Stimulus playbacks are indicated on top (blue: WN, red: rBOS, yellow: BOS). **C** Stackplot of spontaneous activity (no auditory stimulation) in the same ROIs as in **B** reveals that synchronous ΔF/F fluctuations occur spontaneously. **D** Spectra of ΔF/F signals averaged over all ROIs in **B** (black) and **C** (green) with standard error of the mean (SEM) delimited by dashed lines. Both with and without auditory stimulation there are strong fluctuations at low frequencies (<1 Hz).

To study HVC activity on a population level, we produced stack plots of ΔF/F traces in large populations of ROIs. In baseline activity we observed large fluctuations that were synchronized among many ROIs, Figure 6. Fluctuation peaks during inter-stimulus periods often exceeded stimulus-evoked peaks. To assess the influence of auditory stimulation on ongoing baseline activity, we separately analyzed signal co-fluctuations during baseline periods and during auditory stimulation. We defined a measure of synchrony in a particular cell population in terms of the correlation coefficient (CC) between the signal in a given ROI and the mean signal of all other ROIs of the same type.

In HVC_X_ neurons CCs were highest during baseline periods and auditory stimulation had the effect of desynchronizing calcium signals. Indeed, in HVC_X_ neurons at short distances <100 µm, all of BOS, rBOS, and WN stimulation induced desynchronization of calcium signals. CCs were larger during BOS than during rBOS and WN (p<0.001, paired Wilcoxon signed-rank test). Less desynchronization by BOS compared to other stimuli is surprising given that BOS responses were larger than responses to other stimuli. We suspected that baseline HVC activity is somehow supportive of mediating large BOS responses without necessitating concurrent changes in the structure of ongoing correlations. In agreement with this idea, in HVC_X_ neurons we found a strong positive correlation between CCs during baseline periods and CCs during auditory stimulation, implying that ROI pairs with strongly correlated signals during baseline periods also showed strong correlations during BOS. The Pearson Correlation between a) BOS CCs and BL CCs in all HVC_X_ pairs was 0.39 (p = 1.4*10^−62^, N = 1655 pairs), and between b) CCs reporting population synchrony in HVC_X_ neurons during BOS and BL was 0.60 (p = 3.7*10^−23^, N = 226 ROIs). By contrast, in astroglia BOS did not desynchronize baseline fluctuations at all, revealing a qualitative difference in behavior between these two cell types.

### d′ analysis pooled over recording sites (small N analysis)

The observation of correlated HVC population activity reveals that calcium signals in different cells are not independent. To test whether d′ prime statistics remain significant when pooled over recording sites rather than over cells, we performed a stimulation-response analysis on population-averaged signals within each site instead of signals in individual ROIs (i.e., we averaged the simultaneously recorded signals over all ROIs of a given ROI type per site).

For HVC_X_ populations (N = 21 sites in 3 birds) we found that BOS selectivity remained significant but median d′ values of population-averaged HVC_X_ responses were considerably smaller than d′ values across individual cells: median d′ BOS-rBOS  = 0.56 (p = 0.05), median d′ BOS-WN  = 0.91 (p = 0.005). The HVC_X_ population was suppressed by WN (median Z-score  = −0.41, p = 0.003) but responses to BOS and rBOS were not significantly different from zero: BOS (median Z-score  = 0.31, p = 0.23) and rBOS (median Z-score  = −0.19, p = 0.24).

The population-averaged responses in glia (N = 14 sites in 2 birds) revealed BOS preference over rBOS but not over WN: median d′ BOS-rBOS  = 0.56 (p = 0.02), median d′ BOS-WN  = 0.20 (p = 0.9). Z-scores of population-averaged glia responses were positive for all stimuli: BOS (median Z-score  = 0.56), rBOS (median Z-score  = 0.42), and WN (median Z-score  = 0.60).

The population-averaged response in neuropil ROIs (N = 37 sites in 5 birds) confirmed the BOS selectivity across individual ROIs (BOS-rBOS: median d′ = 0.84, p<0.0007; and BOS-WN: median d′ = 0.62, p = 0.02). Also, Z-scores in neuropil populations were positive for BOS (median Z-score  = 0.53), and negative for rBOS (median Z-score  = −0.03) and WN (median Z-score  = −0.25). Thus, in conclusion, when we discounted for correlated responses in neuron and glia populations their stimulus preferences were unchanged.

### Spatial extent of pairwise correlations

**Figure 7:**
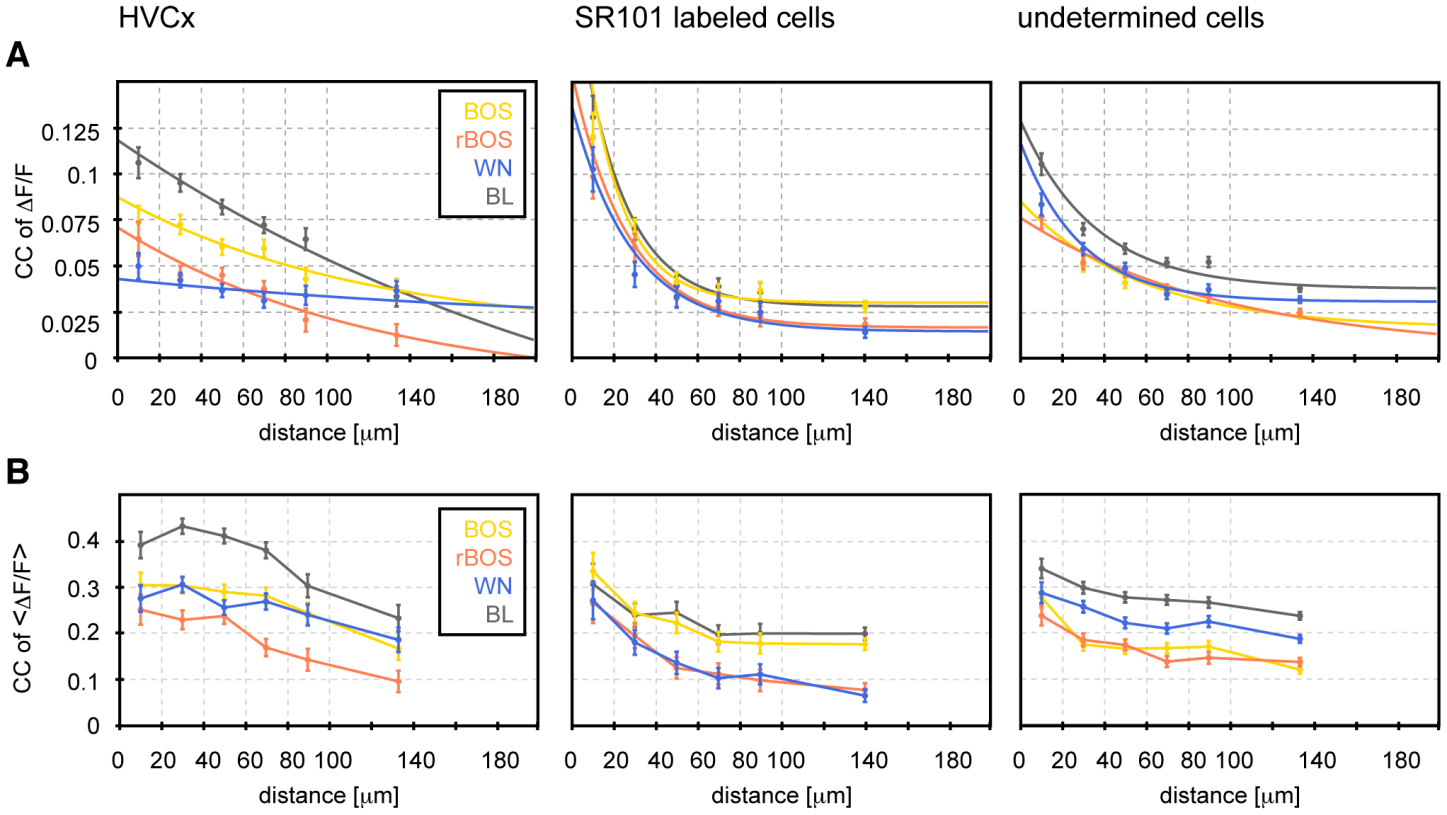
Spatial decay of signal synchrony and response correlation. **A** The average pairwise synchrony (CCs, on the time scale of 0.1 s) in different cell types decreases as a function of the distance between ROI centers. In HVC_X_ neurons the synchrony is strongest during baseline (black) and weaker during BOS (yellow) and even weaker during WN (blue) and rBOS (red). In astroglia the spatial decay of synchrony during baseline and during auditory stimulation are roughly identical. Exponential fits (smooth curves) to non-binned data reveal a large space constant for HVC_X_ neurons (e.g. λ = 263 µm during baseline) and a much smaller space constant for astroglia (e.g. λ = 22 µm during baseline). Distances were sampled in 20 µm bins (dashed vertical lines coincide with bin boundaries). The last bin contains all data points with pairwise distances >100 µm. Error bars are standard errors of the mean (SEM). **B** On a larger 2 s time scale, ΔF/F signals averaged over stimulus periods (3 s windows for baseline - BL) are more strongly correlated but decay more flatly, suggesting slow spreading of calcium signals. In particular, on this timescale, the spatial correlation profile in astroglia resembles that of HVC_X_ neurons on the faster time scale in **A,** with BOS eliciting higher correlations than other stimuli.

We also explored a possible topography of synchronized activity by binning distances between pairs of ROI centers into 20 µm bins and plotting the mean CC versus the distance (Figure 7). We found that CC spatial decay was flatter in HVC_X_ neurons than in glia (neuropil ROIs were excluded in this analysis because of their large spatial extent). We fitted the CC decay with exponential functions and obtained a much larger space constant λ in HVC_X_ neurons compared to astroglia (e.g., during baseline λ_BL_ = 263 µm in HVC_X_ neurons and λ_BL_ = 22 µm in glia). Hence, HVC_X_ neurons maintained correlations over much larger distances than did astroglia.

In most studies, the definition of baseline activity excludes a roughly 2 s window after stimulus offset (to exclude possible reverberations from the preceding stimulus). To do the same, we repeated our analyses of baseline activity by excluding samples within less than 5 s of stimulus offset. This restriction had only a very minor effect on the fitted parameters of our exponential decay model of pairwise correlations (λ_BL_ values changed by less than 1%). Also, all other findings on the decorrelation of baseline activity by auditory stimulation were unaffected by this restriction, revealing little influence, if any, by reverberating activity.

### Auditory Correlations on long time scales

Our CC measure is useful for assessing synchrony on a 120-ms timescale (fluorescence sampling period). To probe for correlations also on the slower timescale of songs (order 2 s), we averaged ΔF/F signals over stimulation periods before computing CCs (resulting in a measure comparable to electrophysiological spike-count correlations). The result is a more modest decline in CC with distance, rendering the steepness of CC decrease in astroglia comparable to that in HVC_X_ neurons on the faster 120-ms timescale, Figure 7 B. Across cells, correlations on the longer 2-s timescale were about 3 times stronger than on the 120-ms timescale, which may be due to both high-frequency noise in fluorescence signals affecting the 120-ms measurements and to slow offsets of calcium transients which accumulate influences from more rapidly evolving spiking activity patterns.

**Figure 8:**
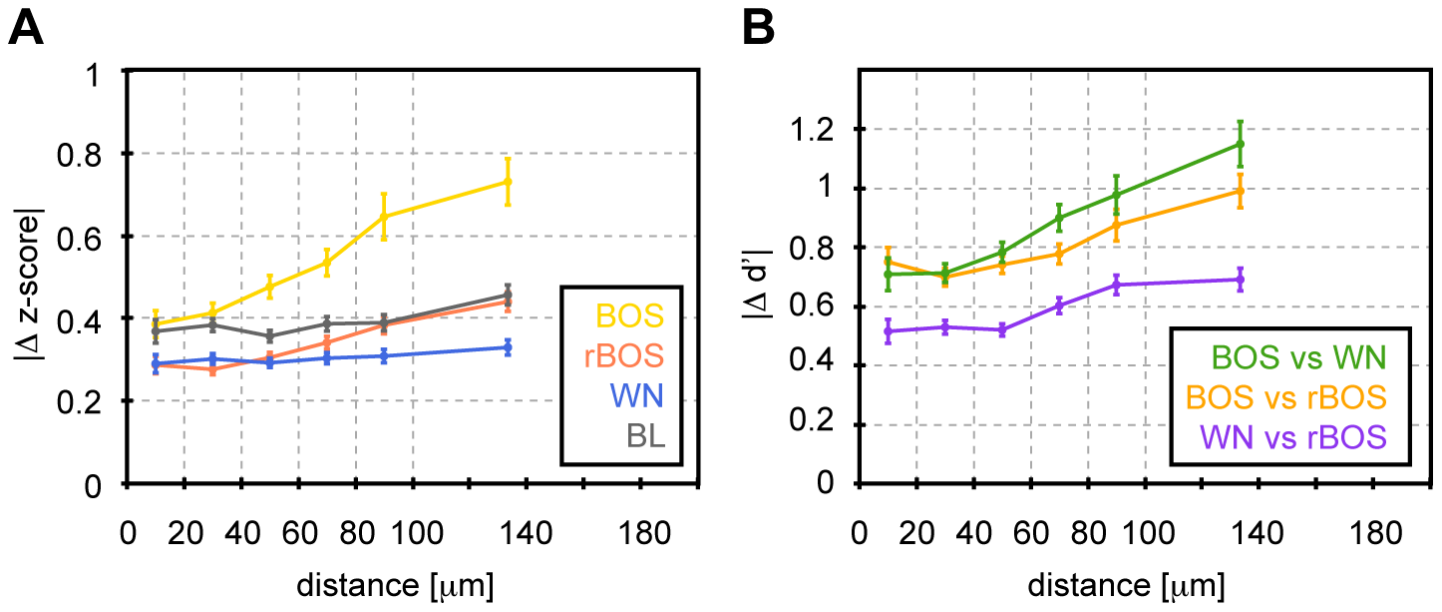
Spatial clustering of auditory responsiveness and selectivity in HVC_X_ neurons. **A** Z-scores for BOS are more similar in nearby cells than in more distant cells. Plotted are absolute Z-score differences as a function of distance between HVC_X_ neurons (ROI centers). Error bars depict the standard error of the mean (SEM). **B** A similar behavior is seen for d′ values that for BOS are more similar in nearby cells than in more distant cells. Distance binning as in Figure 7.

The local nature of synchrony was also partly applicable to measures of responsiveness and selectivity: Nearby HVC_X_ neuron pairs (with distance less than 60 µm) displayed more similar Z-scores and d′ values than did distant neuron pairs, providing evidence of spatial clustering of HVC_X_ neurons with similar response properties (Figure 8). We did not attempt to verify similar trends in glia due to their lack of selectivity to auditory stimuli (Figure 5). Also, because of the significant spatial extent of neuropil ROIs, we did not attempt to evaluate spatial profiles of synchrony and responsiveness in pairs of neuropil ROIs. Nevertheless, neuropil ROIs stood out in terms of synchrony: the average CC in pairs of neuropil ROIs was at least three times higher than that in neurons and glia cells, even when considering pairs of neurons and glia at very short distances (but note that the stronger correlations found for neuropil agrees with a simple model in which fluorescence measurements are described by a common signal and Gaussian uncorrelated noise among pixels).

## Discussion

We have demonstrated the suitability of two-photon calcium fluorescence imaging for studying HVC population responses to auditory stimuli. We discuss the significance of our findings by comparison to results obtained with electrophysiology and in context of the broader literature on dynamics of neural population responses.

We found HVC spikes to trigger rapid rise of OGB-1 fluorescence on the order of tens of milliseconds and much slower decay on the order of one second, Figure 2. Fluorescence signals were mostly non-saturated because when multiple action potentials occurred in short succession we observed the summing of transients in significant excess of transients elicited by single spikes. The temporal resolution of calcium activity in our experiments was on the time scale of song syllables, suggesting that two-photon calcium imaging might offer further valuable insights into sensory coding of HVC neurons, for example to study their temporal combination sensitivity (consisting of supralinear responses to syllables and syllable components when presented in natural sequence order compared to randomized order [41], [42]). Our experiments are also encouraging for studying HVC population activity during relevant behavioral states such as sleep and song production; from electrophysiological recordings in these states we know that projection neuron high-frequency bursts are separated by time intervals on the order of several hundreds of milliseconds and thus may be resolvable by calcium fluorescence imaging.

In contrast to a previous study that investigated topography of HVC auditory responses in zebra finches using serial multi-unit recordings on a coarse ∼200 µm grid [21], we simultaneously monitored neuronal and glial responses on a finer scale of local cell groups. Because we find spatial response topography among neurons at short distances (they are more strongly correlated than more distant neurons), the prevalent view of globally synchronous HVC responses to BOS should be refined by a view that emphasizes this local structure.

Our work extends findings obtained from paired and triplet extracellular recordings in sleeping birds which revealed that spike-train cross-correlation functions from identified HVC neuron pairs agree with a single dynamic variable (encoded globally in HVC) to which single-neuron activity locks probabilistically [43]. The steep spatial decay of pairwise ΔF/F correlations suggests that HVC dynamics are best described not by a global state but by more local states with restricted spatial structure. We did not record from cell pairs separated by more than 200 µm, and therefore it remains unknown whether positive pairwise correlations extend beyond these distances. Given HVC's redundant organization revealed in experiments in which HVC was sectioned into two halves but there was no significant effect on produced song [44], it is likely that HVC sub-networks separated by more than 200 µm are independently organized and that HVC baseline activity is not significantly correlated beyond these distances.

We explored possible auditory specialization in diverse HVC cell populations. In HVCX neurons, responses to BOS were stronger than responses to other stimuli, confirming previous reports [20]–[22], [24]. Both largest response and least desynchronization seen during BOS stimulation could arise in HVCX neurons from similarity of baseline activity and BOS-evoked activity. Namely, the high correlation of CCs during BOS and during baseline in individual ROIs provides support of the idea that patterns of baseline HVC activity in anesthetized birds constitute some form of replay of patterns seen during singing (i.e., correlated cells during baseline are correlated during BOS; together with the known similarity of BOS-evoked activity and song-related activity this suggests that correlated cells during baseline are also correlated during song) [16], [43], [45], [46]. Accordingly, the suppressive effect of rBOS and WN on ongoing activity (reduced Z-scores) could arise from lack of similarity between rBOS/WN responses and baseline activity, whereas the enhancing effect of BOS may be due to similarity between BOS responses and baseline activity. HVC contains a large population of inhibitory interneurons that could mediate suppression of stimulation-evoked activity patterns not matched in structure to spontaneously generated patterns [47], [48].

How can auditory stimulation both enhance responses and suppress pairwise correlations? The interplay in neural populations between strengths of responses and correlations has been carefully studied in the past; there is a well-known monotonic relationship between average firing rates and pairwise firing-rate correlations [49]. However, such relationship cannot explain our findings because HVC_X_ responses during BOS were higher than during baseline periods, based on which we would have expected BOS-associated correlations to exceed baseline correlations, which was not the case. We speculate that auditory stimulation led to reduced correlations because auditory tuning tends to be nonlinear and diverse among HVC cells [50], possibly acting to desynchronize cells. Weak influence of auditory stimulation on pairwise correlations is widespread, such phenomenon has been reported even in sensory areas in which the influence of auditory stimulation on baseline activity patterns can be very small [51], [52].

Neuropil was by far the most strongly synchronized ROI type, which to large extent is due to our neuropil ROIs being much larger than cellular ROIs. Indeed, by shrinking neuropil ROIs down to the size of a soma, neuropil synchrony became comparable to HVC_X_ synchrony. Nevertheless, unlike HVC_X_ neurons, neuropil signals were not uniformly suppressed by WN and thus we suspect that at least a subset of neuropil signals reflected both local intrinsic and afferent inputs from NIf, CLM, and Av: First, the trend of increasing response selectivity along the auditory pathway from weak selectivity for WN in field L to strong selectivity for the BOS in HVC [53]–[56] agrees with increased WN responses in neuropil. Second, the relatively small cell count in the latter areas suggests diverging connectivity patterns onto HVC, in agreement with increased correlations in the input and thus in neuropil.

On average, astrocytic auditory responses were very weak but positive to all stimuli including WN, a property that in our study distinguishes astrocytes from HVC_X_ neurons and from neuropil. Lack of BOS selectivity of astrocytic responses may relate to independence of astrocytic responses on postsynaptic excitatory activity: Astrocytic calcium responses to sensory stimulation have been reported in rat barrel cortex, they depend on synaptic release of glutamate but depend neither on AMPA or NDMA receptor activation [29]. Thus, HVC astrocytic responses may be driven largely from spillover of afferent inputs to HVC that are known to be less BOS selective than are HVC neural responses. Dominance of glutamatergic release from synaptic afferents rather than local HVC circuits also agrees with the sparse firing of HVC projection neurons (being the only known glutamatergic cells in HVC).

HVC astrocyte signal correlations were more strongly localized than were HVC_X_ correlations. Local synchronization of astrocytic activity agrees with reports of astrocytic activity waves in hippocampus [57]. Strongly localized correlations are in line with electrophysiology and morphology: astrocytes do not spike and their arbors extend over much shorter distances than do HVC_X_ arbors (tens of µm in astrocytes versus up to 1 mm in HVC_X_ neurons). Possibly, correlations are driven by communication within networks of similar cell types, i.e. networks among HVC_X_ neurons [58] on the one hand and networks among gap-junction coupled astrocytes [59] on the other hand. Our findings suggest that the limiting factor on correlation strength is the distance in connection space or degree of connectivity among cell types (which is large for distant astrocytes but may be small for distant HVC_X_ neurons).

The function of HVC astrocytic sensory responses is unknown. Astrocytes are hypothesized to be intimately and bi-directionally involved in neuronal signaling and in the process of synapse formation and stabilization; implantation of immature astrocytes can lead to rearrangement of ocular-dominance maps in the cat [60]. Given that in canaries there are seasonal changes in the density of HVC astrocyte networks [30], it may be that such changes are influenced by auditory responses, perhaps paralleling the remarkable structural changes seen in HVC neurons of young birds triggered by exposure to a song model [9], [61].
